# Reflective journal writing and interpreting anxiety: insights from a longitudinal study

**DOI:** 10.3389/fpsyg.2025.1671239

**Published:** 2025-12-04

**Authors:** Zhenzhen Wang

**Affiliations:** School of Foreign Languages, Shanghai Dianji University, Shanghai, China

**Keywords:** reflective journal writing, interpreting anxiety, task anxiety, error anxiety, confidence anxiety

## Abstract

This mixed study explores the impact from reflective journal writing upon students' interpreting anxiety. It draws on data sources from students' reflective journals written during the semester as well as two questionnaires of Anxiety Scale for Chinese Interpreting Learners. The study focuses on three dimensions of interpreting anxiety, namely task anxiety, error anxiety and confidence anxiety. Task anxiety has been significantly alleviated over the course, while the other two have not. The cognitive–affective–behavioral intervention mechanism identified in students' reflective journals, functions effectively in mitigating interpreting anxiety. The study also reveals how instructed reflective journals enable students to perceive themselves as self-motivated and sustainable learners. This study holds important pedagogical implications for educators in language teaching and other educational context.

## Introduction

In the development of higher education, researches have long noticed learning anxiety as a crucial factor affecting students' language performance (Scovel, 1978; Horwitz et al., 1986; MacIntyre and Gardner, 1991a,b; Horwitz, 2001; Woodrow, 2006). Interpreting learning is of no exception. It is a highly complex language performance involving not only language, but also culture, encyclopedia knowledge etc. Researches have shown that stress and anxiety affected students' interpreting performances and interpreting competence development (Cooper et al., 1982; Cai and Dong, 2015). Reflective Journals, employed as an educational aid in some disciplines, have demonstrated the ability to alleviate students' learning anxiety and enhance their academic performance and self-efficacy.

Reflective journals encourage students to record and reflect on their learning process, enabling students to better understand and apply what they have learned. In the meantime, it provides an outlet for emotional release. Previous researches found that reflective journals could significantly reduce the anxiety levels of nursing students during clinical internship. This not only benefited students' learning but also enhanced patient safety (Goodman and Henry, 2019). In the field of language learning, reflective journals alleviated learning stress to some extent by enhancing students' self-awareness and internal leadership (Pavlovich, 2007). The effective use of reflective journals could help students conduct more critical reflections and improve their language learning outcomes (Power, 2012). Through recording in reflective journals, students could rapidly improve their critical thinking skills and gradually reduce their anxiety during the reflection process (Threlfall, 2014). Korean adult EFL learners could reduce their ambiguity anxiety in English learning and improve their course grades to a certain extent through online reflective journals (Pyo, 2023). Some scholars also studied semi-structured reflective journals and found that they had a significant effect on cultivating students' cognitive regulation (Alt and Raichel, 2020).

Although reflective journals does alleviate students' learning anxiety and enhancing learning outcomes as shown in existing research fields, there is a lack of research in the specific area of interpreting learning. Given the high-difficulty and high-stress nature, it is of great practical significance to explore the application of reflective journals in interpreting learning and to evaluate its impact on alleviating interpreting anxiety. This study aims to fill this research gap by discussing the implications of how instructed reflective journals influence students' interpreting anxiety through empirical research.

## Literature review

### Theoretical framework

**Figure 1:**
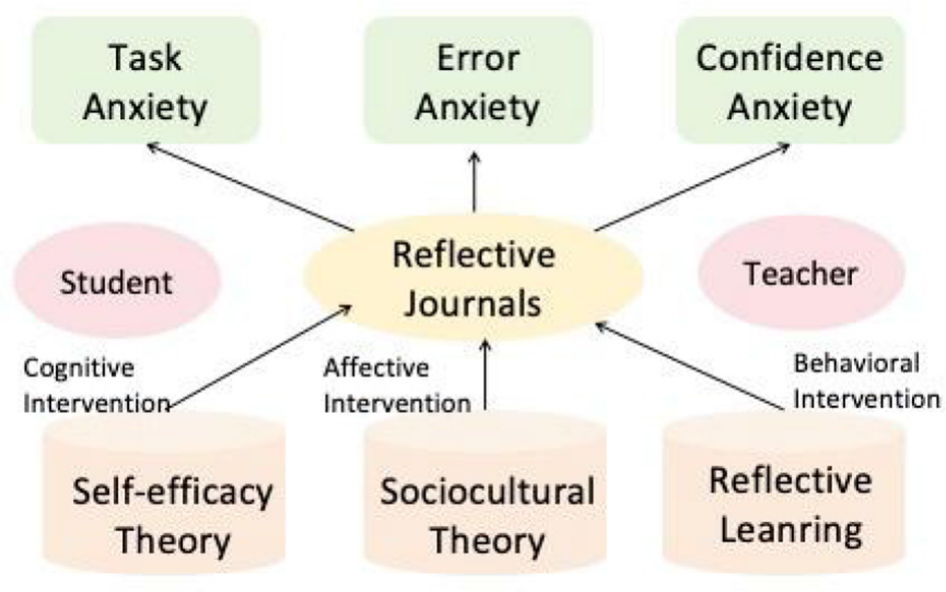
Theoretical framework of “cognitive–affective–behavioral intervention mechanism”.

Regarding the theoretical foundation from reflective journals, this study tries to construct a theoretical framework that integrates multiple perspectives, providing a comprehensive lens through which the “cognitive–affective–behavioral intervention mechanism” of reflective journaling can be understood (Figure 1).

#### Self-efficacy theory: cognitive intervention from reflective journals

Self-efficacy theory, introduced by Bandura (1977), proposed that an individual's belief in their capabilities to perform tasks significantly influenced their emotional responses, including anxiety. Self-efficacy was directly linked to students' ability to face challenges with confidence rather than avoidance or fear. Bandura's theory provided an essential foundation for us to understand how reflective journals enabled students to recognize their own progress and successes in interpreting. As they reflected upon their performances, they acknowledged small wins and developed a clearer sense of their own abilities, through diminishing the cognitive distortion that often fuels anxiety. Therefore, it was safe to say that cognitive intervention from reflective journals helped develop a growth mindset—seeing challenges as well as opportunities to improve rather than obstacles that induce anxiety.

However, this was not just a process of self-reflection; it was a process of self-reinforcement. Through journals, students internalized positive feedback about their progress and, as Bandura (1977) explained, mastery experiences (previous successes) contributed to higher self-efficacy. As their belief in their own competence grew, so did their ability to handle the pressures of interpreting, leading to decreased anxiety levels.

#### Sociocultural interaction: reflective journal as an affective intervention process

While self-efficacy was critical, it did not occur in isolation. Sociocultural theory, as articulated by Vygotsky (1978), emphasized the social context in which learning took place. Vygotsky's concept of the zone of proximal development (ZPD) suggested that learners achieve greater cognitive development through interaction with more knowledgeable others—teachers, peers, and mentors—who scaffolded their learning. We found this concept to be particularly useful in reflective journals, which served as an affective medium for gaining socially interactive learning.

Reflective journals did not occur in a vacuum. When integrated with peer feedback or teacher commentary, students could engage in a deeper processing of their emotions and learning experiences. Vygotsky's theory implied that reflection was not just an individual endeavor but was enhanced by social interaction and feedback. Under our study of interpreting context, students would experience anxiety most frequently because of the perceived isolation between their tasks and the uncertainty about their abilities. Once they received feedback, either from peers or from teachers, upon their reflections, they were able to re-frame their experiences and confront their anxieties with a greater sense of support and understanding. Thus, reflective journals allowed for the integration of scaffolding and affective support, helping students gradually reduce anxiety through both introspection and social validation.

#### Reflective learning: from experiential reflection to behavioral intervention

John Dewey's reflective learning theory (1933) provided an additional layer of insight into how reflective journals could alleviate anxiety and improve self-efficacy. Dewey's theory focused on the importance of reflection in learning, emphasizing that reflection was not simply about looking back at experiences but actively engaging with them to derive meaning and inform future action. Reflection led to more purposeful learning because it encouraged learners to question, analyze, and critically evaluate their experiences in behavioral implementation. These qualities fit in just well in our study context.

In the context of interpreting, students often faced anxiety because they felt uncertain about how to perform under pressure or handle unexpected challenges. Dewey's framework suggested that through reflection, students not only analyzed their emotional responses but also formulated concrete strategies for improvement. During our study, the act of writing reflective journals allowed students to process their feelings of inadequacy or nervousness, understand the root causes of these emotions, and adjust their future approaches to interpreting tasks. Thus, the process of reflective thinking became a form of cognitive and emotional regulation, helping students re-frame their anxiety and shift toward more constructive, self-assured behaviors in subsequent interpreting tasks.

By integrating these perspectives, we can develop a unified framework (see Figure 1) to explain how reflective journals exert a positive impact upon students.

#### Alleviating students' anxiety: a historical overview

Dewey stressed that reflection was an active and continuous careful consideration of the sources of beliefs and knowledge and regarded reflective thinking as a vital element in education (Dewey, 1910). During the process of writing reflective journals, students deepened their understanding of learning content and self-awareness, ultimately improving their learning effectiveness (Power, 2012).

### Reflective journals

Reflective journals were first applied in the research on teacher development in the educational field, emphasizing that teachers independently explore methods suitable for their professional growth (Vujnovic and Medic, 2022). Reflective journals have been widely used in various educational contexts. Some researchers emphasized the varying emotional regulation effects of reflective journals at the individual level (Goodman and Henry, 2019). Some empirical studies showed that reflective journals contributed to the development of meta-cognition (Alt and Raichel, 2020), and writing reflective journals could be an important strategy for enhancing college students' meta-cognitive abilities (Bu, 2022).

### Alleviating anxiety in students' learning

The application of reflective journals could significantly alleviate students' learning anxiety and promote mental health. Threlfall (2014) and Pavlovich (2007) both emphasized the positive impact of reflective journals on students' self-awareness and emotional regulation. Lothes et al.'s (2021) research explored the alleviation of college students' test anxiety and the improvement of learning efficiency through mindfulness-based reflective diaries. Brown et al. (2022), through a systematic review, found that reflective journals are effective in promoting the reflection and the development of clinical judgment and emotional abilities of nursing students. However, they also pointed out that further research is still needed to develop effective assessment tools to better measure the specific effects of reflective journals.

### Alleviating anxiety in language learning

In language learning, the use of video-based reflective resources provided insights into alleviating students' anxiety in oral communication, listening and business English learning classes. (Villamizar and Mejía 2019) indicated that the use of digital video reflective journals could significantly improve college students' language expression ability and reduce their anxiety in oral communication. Similarly, Cinkara (2016) used the video-stimulated recall technique to prompt language learners to reflect on oral production tasks and found that stimulated-recall reflection helped students notice and reflect on the symptoms and causes of classroom anxiety.

These studies also provided formative evaluation on the perception of learners' abilities. Through data from reflective journals and interviews, some researchers discovered the advantages of video-based self-evaluation. Empirical research on language learning also demonstrated that reflective journals contributed to enhancing students' abilities in listening reflection and positive emotional traits in critical thinking (Ren, 2018). They were helpful for cultivating the critical thinking awareness of students in business English study (Zhu, 2021). The effectiveness of reflective journals in alleviating language learning anxiety paved solid foundation for this study.

### Alleviating anxiety in interpreting learning

In the field of translation/interpreting, entries in students' reflective journals had been effective in the evaluation of translation competence development (Wu, 2014) and the interaction between teacher and students in interpreting teaching (Yao, 2014). Wu's study focused more on the composition of students' translation competence and the subsequent impact upon pedagogical practice. Yet Yao sorted out the difficulties proposed in students' reflective journals, most of which involved a certain dimension of interpreting anxiety. He stressed the importance of action research in collecting and analyzing reflective journals during interpreting course. However, relevant research is still scarce, and the specific application research of reflective journals in interpreting learning remains limited. Based on the previous studies, this study finds it necessary to further the study in reflective journals and its mechanism in alleviating interpreting anxiety.

Therefore, this study intends to address the following two questions:

(a) In what dimensions do reflective journals influence students' interpreting anxiety?
(b) How do reflective journals help alleviate students' interpreting anxiety?

By answering these questions, it is expected to enrich the empirical research on reflective journals in the field of interpreting learning and to provide effective references for interpreting teaching.

## Method

**Figure 2:**
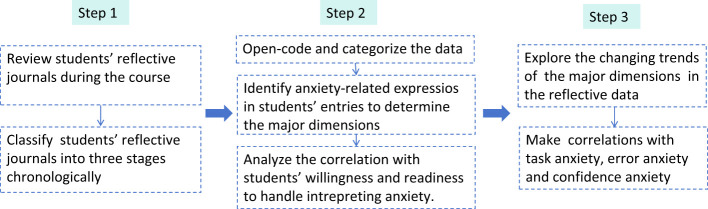
Qualitative data analysis procedure.

This study adopted a mixed-research method that combines qualitative and quantitative research to achieve the research objectives. The qualitative research mainly focused on students' reflective journals within the semester (from Sep. 2024 to Jan. 2025). Entries of students' journals were reviewed and classified into three stages chronologically. Data from these three stages were carefully coded through open coding and *in vivo* coding on MAXQDA 2020 to mark the anxiety-related expressions (see Figure 2 for the data analysis procedure).

For the quantitative research, this study adopted the Anxiety Scale for Chinese Interpreting Learners by Dong et al. (2013). The scale was developed to include 20 items to assess interpreting anxiety for interpreting trainees in China, such as the enormity of the task, the service nature of the task, general confidence, etc. The scale was issued to students at the initial stage and also at final stage of the semester to record the growing trend of their interpreting anxiety. The quantitative results also provided certain verification for the qualitative data.

### Qualitative research methodology

#### Setting and participants

The participants for this study were 47 students (14 male and 33 female) English majors at a local university in Shanghai. These 47 students were in their third year in college. All of them had already passed College English Test Band 6 before taking the interpreting course, hence their language competences were at the approximate level.

During this “English-Chinese Interpreting” course, students were asked to complete the reflective journals independently after class at the end of each week. Altogether participants handed in 9-weeks' reflective journals during the semester, 363 journal entries in total. This research was approved by the college ethics committee and participants were assured that no specific identifying information about the course would be processed.

#### Format of reflective journals

To collect data on students' interpreting learning process in a targeted manner, the teacher explained the significance and purpose of writing interpreting reflective journals in the first week of the semester. Students were clearly informed that the reflective journals of this semester were mainly used for the analysis and diagnosis of this particular course, and that reflective journals were written on a voluntary basis with no word-count requirements. Considering students' language abilities and the effectiveness of reflection, it was recommended that students write the journals in their mother tongue, here in Chinese.

The teacher also explained the writing format of the reflective journals, based on the six stages of “Description–Feeling–Evaluation–Analysis–Conclusion–Action”, as is suggested in Gibbs' structured reflection model (Gibbs, 1988).

Gibbs' model was initially used in the nursing field to observe students' clinical performance (Taylor-Haslip, 2010) and the development of their critical thinking (Barnes, 2010). Scholars have also applied this model to literary analysis (Adeani et al., 2020) and EFL teacher reflective writing (Hashim et al., 2023). Gibbs' reflection cycle emphasized understanding and improving behavior through reflection (Gibbs, 1988). Its cyclic nature coincides with the characteristic of interpreting teaching that emphasizes repeated practice. Moreover, the stage goals and guiding questions in the reflection cycle are helpful for students to analyze systematically and to reflect effectively on the interpreting learning process.

This study adapted the six-stage model and integrated it into the instructed theme prompts of interpreting reflective journals (see Table 1). Since the “conclusion” and “action” stages indicated both the retrospective quality and the required action regarding it, these two stages were combined together to represent the last theme in the format prompts.

**Table 1:** Format of reflective journal and its correlation with Gibbs' reflection model.

** 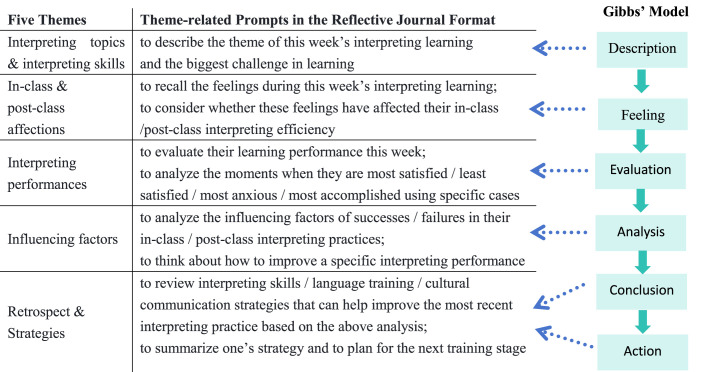 **

Before the initial writing experience, the teacher explained specifically five theme-related prompts in the reflective journal format, namely, the writing subjects, ways of reflection and how to record authentic personal actions and emotions during writing etc.

During the reflective journal writing process, students began with “describing the theme of this week's interpreting learning and the biggest challenge in learning”. Then, they “recalled the feelings in this week's interpreting learning” and “considered whether these feelings have affected their in-class/after-class practice effects”. Moreover, they “evaluated their learning performance this week, and analyzed the moments when they were most satisfied/least satisfied/most anxious/most accomplished with specific cases”. They were also encouraged to “analyze the influencing factors of success/failure in their in-class/after-class interpreting practice” and “think about how to improve a specific interpreting performance”. Based on the previous steps, students were able to “derive interpreting skills/language training/cultural communication strategies that could help improve the effect of the most recent interpreting practice”. Finally, they could “formulate the next learning strategy” and “plan the training goals for the next stage”.

### Data collection

It is noted that before writing the reflective journals, students were well aware that they would process with such perspectives as description, feeling, evaluation, analysis, conclusion, and action. They could choose to describe in detail or in general based on their specific learning status quo of each particular week. A series of actions such as summarizing, questioning, planning and rethinking were involved before they finally recorded the above-mentioned activities in the form of reflective journals–somewhat similar to the action of “think-aloud” (Gerloff, 1986). Their reflective journals were sent to the teacher's email within 3 days after the class.

At the same time, the teacher completed the feedback before the start of a new week's session. The teacher sorted out their reflection journals, marking the homogeneous problems raised by students for further explanations in the next class session, selected some useful methods, new thoughts, or implementable plans from the reflective journals and asked some journal writers to share with the whole class the next week, of course with their consent.

**Figure 3:**
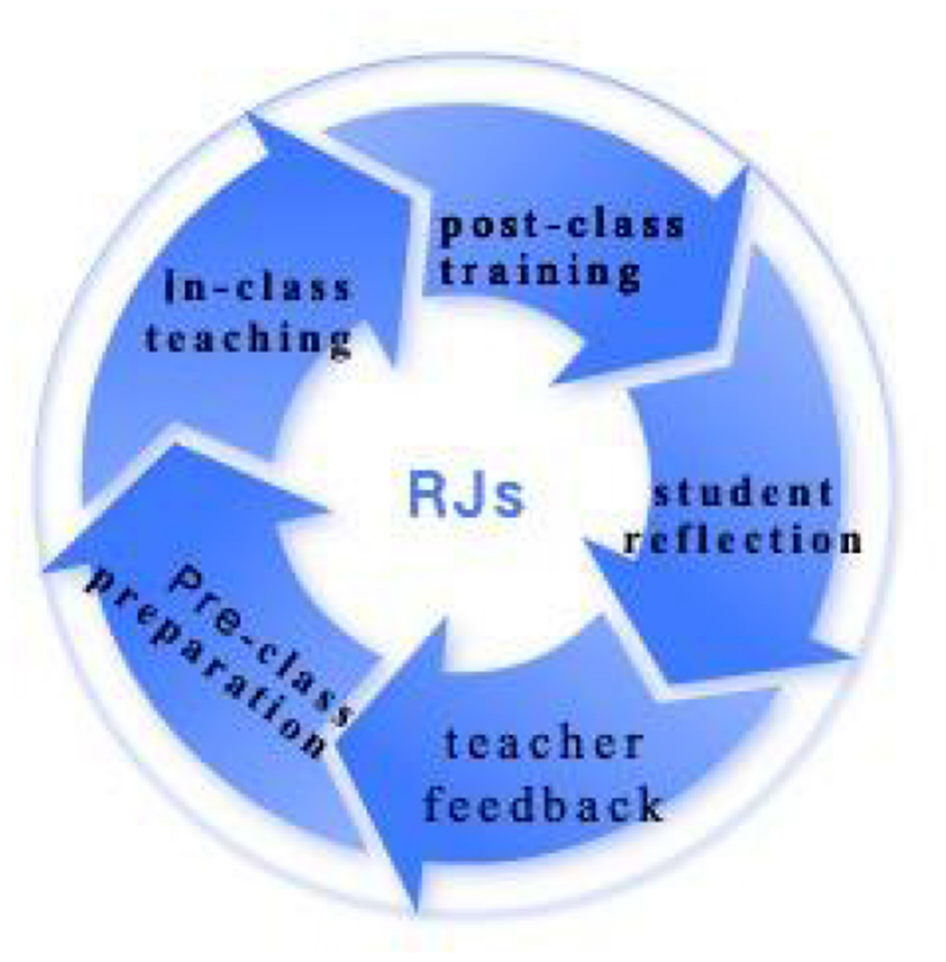
Closed loop for interpreting course adopting students' reflective journals.

In this way, teacher and the students together formulated a favorable mini-educational ecology of closed loop in interpreting course, as is shown in Figure 3. It starts from in-class teaching to post-class training, students' reflection, teacher feedback and finally pre-class preparation for new interpreting tasks for the next week.

### Quantitative research methodology

Quantitative data were collected to see the overall development of the interpreting anxiety of the same group of students during this specific semester. The interpreting anxiety scales were administered to the participants, both in the initial stage (Week 3) and in the final stage (Week 15) of the semester. Before obtaining students' consent, it was specified that the questionnaires were anonymous and that no pressure would be applied should their choose to leave the questionnaire unfilled or half-filled. The research was approved by the college ethics committee.

The questionnaire used was a scale developed by Dong et al. (2013) based on the learning scales for Chinese interpreting students. It consists of 20 questions, using a 5-point Likert scale. This scale has been tested to have good internal consistency reliability, construct validity, predictive validity, and test-retest reliability. It has been proved to be suitable for investigating the changes in interpreting anxiety of interpreting trainees.

### Findings

#### Qualitative results

In this study, students' reflective journals collected were divided into three stages chronologically: the initial stage (including week 3, 5), the mid-semester stage (including week 6, 7, 9, 10), and the final stage (including week 11, 12, 15). Students' reflective journals were classified and coded based on the three phases of the semester. Data was carefully analyzed to observe the trajectory changes over the course. It allowed researchers to identify the influences upon students' anxiety dimensions and levels and to explore how these influences actually took effect resulting from instructed reflective journal writing.

A detailed analysis showed that under the main theme of “anxiety”, three major dimensions, namely task anxiety (TA), error anxiety (EA), and confidence anxiety (CA), were identified almost in every weekly reflective journal entry.

For example, one student (S36 was chosen randomly) recorded:

“*My hand couldn't follow my mind and I find it hard keeping down the key words*. [TA] *I fell into complete chaos and disaster*.[CA]*......Often I regret about having not remembered some key words and sometimes basic knowledge, even common sense would fail me. That was really frustrating! The more I got stuck, the more I was afraid of making mistakes and not translating the meaning accurately*[EA]. *I kept saying ‘emmm' and ‘and'.......”* (S36′s entry in week 3)

“*More skills are added in now, like the logic analysis and long discourses. I have to deal with materials with intensive information chunks*.[TA] *Seems like I have to improve my logic recording system and make note-taking my own style and stop copying others.......I think I am doing pretty all right as long as it does not involve some culture-loaded words and terminologies*.[EA]......*I'm not sure about how to deal with some speech styles, especially public speeches*.......[CA]” (S36′s entry in week 10)

“*I've just finished interpreting the speech about Chinese sports and Wushu. It was really a disaster*.[CA] *The passage is so so so long with a lot of cultural words and also the four-character phrases, It requires so much as of visualization and logical organization and of course your handle of traditional Chinese culture*.[EA] *My brain just drained out*.[CA]......*I need to practice more in the similar topic since I myself am not that interested in sports. Maybe a mini-corpus may be of help*.” (S36′s entry in week 12)

For this specific example which was randomly drawn, task anxiety, error anxiety and confidence anxiety were spotted in each entry in the three stages of the semester. The discussion of any dimension could not be escaped from the other two. Also, with a detailed reading of S36′s reflective journals during a longitudinal period, it was clear that task anxiety was alleviated greatly through reflective journals. Yet, error anxiety and confidence anxiety were relieved mildly and still kept at a relatively high level.

**Figure 4:**
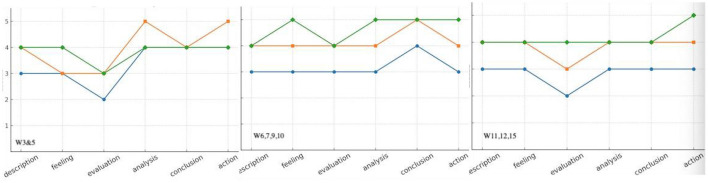
The longitudinal changes from task anxiety, error anxiety and confidence anxiety in S36′s reflective journals. Blue, task anxiety; Orange, error anxiety; Green, confidence anxiety.

Figure 4 showed that in the long run, task anxiety experienced a stable decrease and kept at a relatively low level for a long period in all the six stages in Gibbs' model. It went at the lowest level at the stage of “evaluation” in both the initial stage and the final stage of the semester. Error anxiety was comparatively at a higher level than task anxiety with several fluctuations. It reached its peak at the stage of “analysis”, “conclusion” and “action”. Confidence anxiety was, most of the time, at the highest level among the three with a growing trend and rose to its peak level in the final stage of the semester.

**Figure 5:**
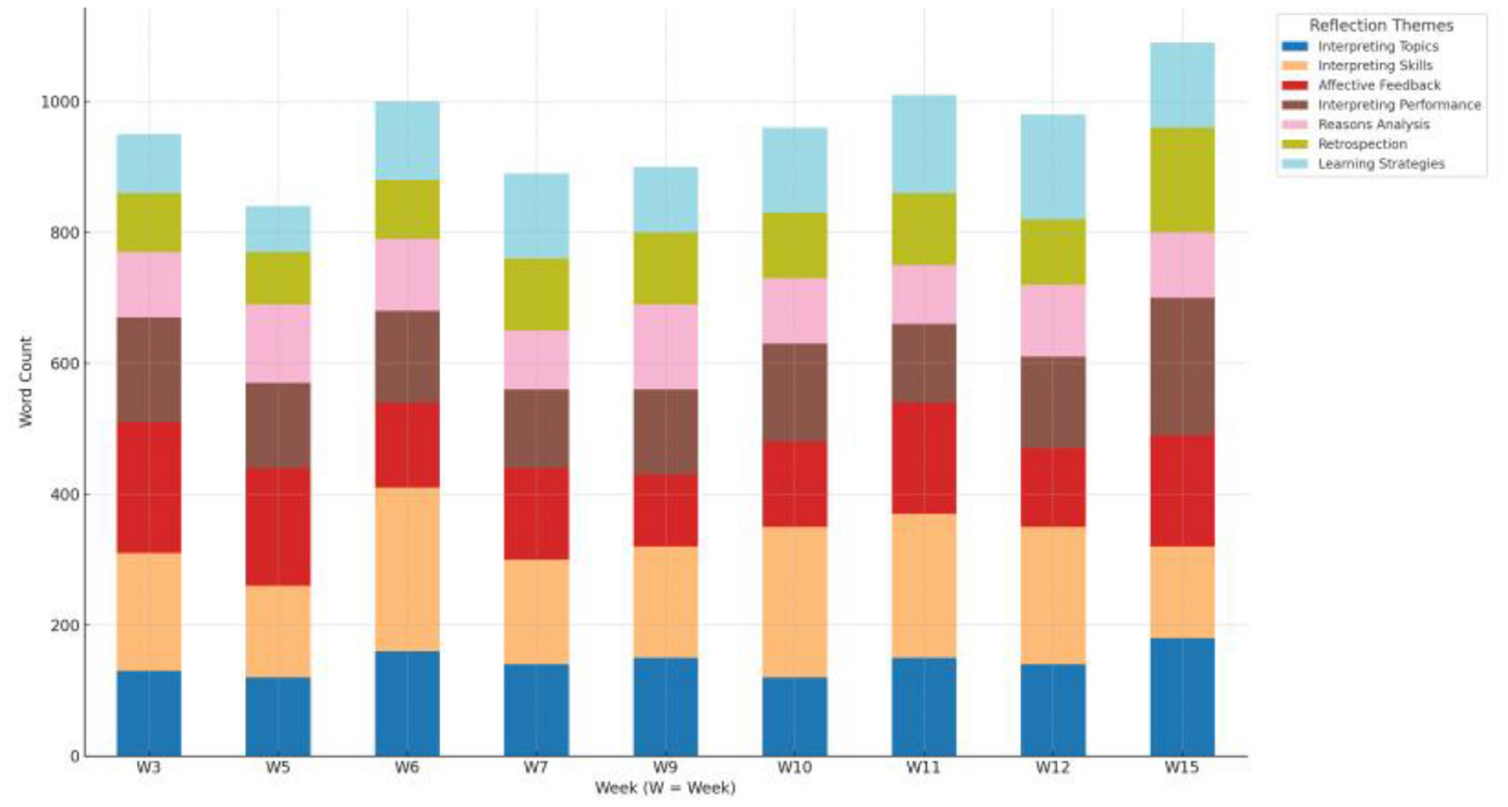
Word-count from the reflective themes in S36′s reflective journals.

Furthermore, word-count usually represents the participant's willingness to discuss a certain topic he/she encounters, which in return influences his/her readiness to make individual efforts to lessen interpreting anxiety. When words in S36′s journals were counted based on the instructed reflective themes (see Figure 5 for word-count for reflective themes), it was noted that he spent the most efforts each week reviewing theme relating to “Interpreting topics and skills” after class, especially in mid-semester stage (week 6, 10, 11). It indicated his deeper understanding of individual interpreting practice with the passing of time. Discussion on the theme of “Learning strategies” became more frequent especially after week 10 regarding topics like improving information organizing skills, dealing with intercultural problems linguistically etc. This indicated a gradual rising consciousness of self-efficacy. Reflective theme like “Affective feedback” was stable all over the time, recording his real-time feelings. In the final stage (week 9 and 12), reflective theme like “Reason analysis” was most conspicuous, exploring details about interpreting difficulties, mis-interpretations and blindness in encyclopedia knowledge. The most word-count came in the final stage (week 15) (1,090 words in total), when the whole class was devoted to group project of simulated conference interpreting.

#### Quantitative results

The questionnaire issued both in the initial and final stages of the semester respectively was the one developed by Dong et al. (2013) with 20 questions altogether. It was noticed that most of the questions align with dimensions of task anxiety, error anxiety and confidence anxiety. To make the evaluation easier, Likert Scale was applied to the collected data (“A = 1, B = 2, C = 3, D = 4, E = 5”). Yet for Question 2, which adopted converted textual responses to numerical scores, a reversed ranking system was applied correspondingly (“A = 5, B = 4, C = 3, D = 2, E = 1”).

Data from pre-survey and post-survey was run by SPSS 16.0 to probe longitudinal changes in students' anxiety levels from the three dimensions and to explore the Pearson correlation coefficient among the three. Statistics showed that the p-value for task anxiety is 0.043 ( ≤ 0.05), much less than the significance level, so the impact upon task anxiety was considered statistically significant. The p-value for error anxiety and confidence anxiety was 0.292 and 0.403 respectively (both >0.05), so the result was considered not significant (see Table 2).

**Table 2:** Longitudinal overview of three anxiety dimensions for pre-survey and post-survey.

**Dimensions**	**Corresponding questions (Q)**	**Mean score (pre)**	**Mean score (post)**	***t*-value**	***p*-value**	**Significance**
Task anxiety	Q2, 12, 13, 15, 16, 17	3.47	3.20	2.07	0.043	^*^
Error anxiety	Q9, 10, 11, 14	3.67	3.51	1.07	0.292	ns
Confidence anxiety	Q18, 19, 20	2.95	2.81	0.84	0.403	ns

^*^means significant.

Table 3 showed the Pearson Correlation Coefficient between task anxiety and error anxiety and that of task anxiety and confidence anxiety were both strong (from 0.71 to 0.76). The correlation between error anxiety and confidence anxiety was relatively strong, very close to 0.50. The quantitative data corresponded to the qualitative data of reflective journals and explained the reasonability of categorization of these dimensions. As the processing of entries from student S36′s reflective journals illustrated, the three dimensions of interpreting anxiety were identified from the initial stage to mid-semester, and all the way to the final stage of the course. Moreover, interpreting entailed, in essence, elements for public speaking. The more anxious one felt during a public performance, the more mistakes one tended to make, which might in return cause more nervous feelings and affect his/her confidence. This also prioritized the significance of alleviating task anxiety in the process of interpreting teaching and learning.

**Table 3:** Pearson Correlation Coefficient for pre-survey and post-survey.

**(Pre-survey)**	**Task anxiety**	**Error anxiety**	**Confidence anxiety**
Task anxiety	1.000	0.714	0.710
Error anxiety	0.714	1.000	0.498
Confidence anxiety	0.710	0.498	1.000
25-1,15.2**(Post-survey)**	**Task anxiety**	**Error anxiety**	**Confidence anxiety**
Task anxiety	1.000	0.712	0.763
Error anxiety	0.712	1.000	0.461
Confidence anxiety	0.763	0.461	1.000

Table 2 illustrated the perceived changes in the three dimensions from a longitudinal overview. The mean score for task anxiety decreased significantly, though it was still spotted in the post-survey. The mean score for error anxiety and confidence anxiety also dropped, but not so much as to be statistically significant. Scores also indicated that for task anxiety, the major distribution came from “unpreparedness”(Q15) and “public performance”(Q12), leading to 77% and 68% of the anxiety respectively. For error anxiety, it mainly came from the fear of forgetting old information (Q14) with a high percentage of 75%, as many were worried that mistakes in memorizing might lead to major errors in interpreting. Confidence anxiety was caused most of the time by inappropriate comparison when one felt extremely timid after observing an excellent classmate interpreting (Q19). 61% of the students were affected by peer pressure. The statistical data here corresponded to the previous qualitative data analysis. The longitudinal trend for task anxiety identified within journal entries also experienced a similar decrease and kept at a relatively low level for a long period over the semester (see Figure 4). It was also worth noticing that word-count for such reflective themes as interpreting topics, skills and strategies was recorded the most (see Figure 5), indicating students' willingness and readiness to tackle corresponding dilemma. Students' efforts made through diligent preparation and deliberate practices worked collaboratively and precisely in tackling such dilemma as had been caused by the high percentage of “unpreparedness”(Q15) and “public performance”(Q12).

## Discussion

Upon the previously presented questions, this study has three findings. Firstly, task anxiety, error anxiety and confidence anxiety are closely correlated during students' interpreting learning process. Task anxiety has been significantly alleviated over the course of the semester. Secondly, the cognitive-affective-behavioral mechanism works actively in students reflective journal writing. It mitigates students' interpreting anxiety, especially in class activities. Thirdly, instructed semi-structured reflective journals function effectively. It enhances students' performance achievement, elevating their self-efficacy.

Both the qualitative and the quantitative data suggest that reflective journals written during the interpreting course have exerted positive influence upon task anxiety, error anxiety and confidence anxiety in interpreting. These dimensions of interpreting anxiety are not selected randomly, but rather based on the strong correlation among them. These data are in line with previous study of interpreting anxiety, since interpreting activity in essence is a public performance of one's linguistic ability, intercultural techniques, knowledge profoundness and feasibility in communication emergencies. This study, suggests that unlike translation in the written form, a negative response in interpreting tasks is likely to be spotted immediately and to induce worries over mistakes and intimidate one's self-confidence.

A longitudinal trend in task anxiety suggests a significant decrease from the initial stage to the final stage of the semester. This study confirms that unprepared interpreting activities account for the major source of task anxiety in interpreting. We also find through data analyzing that with the passing of time, students' accumulated preparation in interpreting skills, topic information and public performance training has been functioning effectively as an outlet for task anxiety.

Students' reflection in their journals follow the “cognitive-affective-behavioral intervention mechanism”. This can be explained through the semi-structured format when Gibbs' reflection model is applied in initiating the theme-related prompts for instructing students' reflective journal writing. Prompts in “description” and “analysis” stages exhibit “cognitive intervention” from reflective journals, while prompts in “evaluation” and “feeling” stages show “affective intervention”, and prompts in “conclusion” and “action” stages demonstrate “behavioral intervention” respectively. The present study, therefore, provides evidence for how reflective journals help cultivate students' self-awareness in interpreting learning.

One way to account for the cognitive intervention is to refer to the reflective journals in such themes as “interpreting topics and skills” and “reason analysis” (the “description” stage). Students are actually keeping tangible cognitive recordings of their hands-on experience in preparation work, language transformation and skills practice, which altogether find ways in enhancing their impression of self-performance accomplishment. It corresponds to what Bandura (1977) identified as one of the four sources of self-efficacy, namely performance accomplishments, vicarious experience, verbal persuation and emotional arousal.

Similarly, evidence for affective intervention is recorded, when students try to evaluate “interpreting performances” (the “evaluation” stage), not just of their own, but also of other students. They benefit from observing and recording peer performances as of vicarious experiences. Affective feedback keeps a stable growth based on the word-count in students' reflective journals, demonstrating real-time emotional recordings and development, in correspondence to what Bandura (1977) called emotional arousal. This study proves that positive peer interaction and model illustrations in class can inspire positive affective power and help boost self-efficacy. Teacher's weekly feedback works collaboratively. Reflective journals can work as the interactive media between teacher and students, helping them cross the “zone of proximal development” (Vygotsky, 1978).

Additionally, regarding behavioral intervention, students' reflection on “learning strategies” (the “conclusion” and “action” stages) is recorded to be more frequent particularly after week 10. This is because from the mid-semester stage, more interpreting skills and themes are introduced into class. Tasks become more and more complicated. Topics range from balancing skills of listening, note-keeping and memorizing, to specific ways of dealing with cultural conflicts. The analysis of the data in the present study indicates that prior successful achievements and valuable feedback help formulate future expectations and consolidate students' persistence on the positive side. The goals and schedules students have planned are now put into live practice. This study echoes previous studies in that the cognitive-affective-behavioral intervention from reflective journals enhances students' self-motivation in language learning.

Last but not least is the semi-structured reflective journal format adopted for instructing students' journals in interpreting learning. This study proves that precisely because of the instructed format, students are able to reflect more thoroughly and more effectively. They can relate individual experiences under the guiding prompts, avoiding reflections that merely stay at the description level or fragmented individual comments. It is conductive in expanding both dimensions and depth of reflections (Waggoner-Denton, 2018). Based on the prompts, students are able to explore their learning scenarios from multiple dimensions. For example, prompts relating to interpreting skills help students in detailing their progress and deficiencies in skills such as logical listening and note-taking practices. Prompts concerning affections facilitate students in exploring the impact of nervous emotions during interpreting. Prompts about learning strategies instruct students in summarizing useful techniques like terminology memorizing and figure interpreting. Prompts on cross-cultural communication guide students in investigating cultural emergencies and coping strategies. After fulfilling all the theme-related prompts, it is convenient for them to ponder over areas for further improvement.

It's worth noting that reflective journals may also increase the learning anxiety of some students and trigger negative emotions. A small number of students may experience negative emotions such as frustration and self-blaming. When they look back on earlier mistakes and deficiencies, unsuccessful memories in the interpreting process might be triggered. Those negative emotions can intensify some students' anxiety, especially for those who are already weak in self-confidence and sensitive to errors during interpreting activities. If these negative emotions are not handled properly in a timely manner, students may fall into a vortex of self-denial. At the same time, writing reflective journals itself may also increase some students' learning pressure. They may worry that their reflections are not good enough to be confirmed by the teacher or think that reflection itself is an additional learning burden. Especially for students with intense learning pressure and psychological fragility, the extra writing practice may intensify their anxiety. This offers warning for teachers to handle the reflective journals with great caution, taking into consideration individual factors as well as the overall pedagogical assignments.

## Conclusion

This study provides empirical supports for positive impacts from reflective journals upon students' interpreting anxiety. Among the three anxiety dimensions, the longitudinal impact upon task anxiety is significant, while the impacts on error and confidence anxiety prove to be mild. In the long run reflective journals function favorably in relieving students' interpreting anxiety through the “cognitive-affective-behavioral intervention mechanism” and semi-structured reflective journals with theme-related prompts have been proved to be effective.

These findings provide strong support for instructors to encourage reflective activities in interpreting course while stressing the importance of peer interaction and positive teacher feedback. Findings of this study also suggest that in interpreting field as well as in other educational contexts, in-time recording of learning activities, retrospection over individual performance, analysis of current learning difficulties and working strategies should be modeled and encouraged. Those pedagogical activities facilitate a favorable mini-ecology of a closed loop such as the “teacher–reflective journals–students” system in interpreting course.

This study used a semi-structured reflective journal format to help students focus on their own viewpoints and the understanding of interpreting learning activities, aiming to build a scaffold for assisting students' meta-cognition (Tripto et al., 2016). Although the semi-structured reflective format provides targeted guidance for students to carry out effective reflection activities, it also has certain limitations. For example, students may be restricted by the existing format and unable to discuss other themes not included on the format. In addition, when some review interpreting activities, the sense of frustration and pressure generated by unhappy retrospection increases the degree of anxiety, and writing reflective journals itself also brings certain task pressure to some students. Still, we can not neglect the fact that while task anxiety is significantly alleviated during the course, error anxiety and confidence anxiety are not. More work needs to be done to include psychological study and interactive approaches.

At the same time, it should be noted that it is difficult to alleviate interpreting learning anxiety within a short period of time. The diachronic research period of reflective journals can be extended to 1 year or even longer, or horizontal comparisons of reflective journals in different grades and classes can be carried out. It is hoped that more data can be collected to provide more implications for interpreting pedagogy.

## Data Availability

The original contributions presented in the study are included in the article/supplementary material, further inquiries can be directed to the corresponding author(s).
